# Aminochrome-Induced Disruption of Autophagosome-Lysosome Fusion: Implications for Protein Aggregation in Parkinson’s Disease

**DOI:** 10.3390/antiox15060739

**Published:** 2026-06-10

**Authors:** Andrea Briceño, Cipriano Núñez, Karina Cortés, Patricia Pallacán, Nicole Salinas, Carola Millán, Juan F. Vivanco, Nelson Caro, Juan Segura-Aguilar, Irmgard B. Paris

**Affiliations:** 1 Centro de Investigación Austral Biotech, Departamento de Ciencias Básicas, Facultad de Ciencias, Universidad Santo Tomás, Viña del Mar 2561780, Chile; ciprianonp@gmail.com (C.N.); karina.cortes.20@gmail.com (K.C.); patriciabrito.pallacan@gmail.com (P.P.); nicole.salinaszarate@gmail.com (N.S.); nelsoncarofu@santotomas.cl (N.C.); 2 Molecular and Clinical Pharmacology, Instituto de Ciencias Biomédicas (ICBM), Faculty of Medicine, University of Chile, Independencia 1027, Santiago 8380453, Chile; andrea.briceno.u@gmail.com (A.B.); jsegura@uchile.cl (J.S.-A.); 3 Facultad Artes Liberales, Universidad Adolfo Ibáñez, Viña del Mar 2580335, Chile; carola.millan@uai.cl; 4 Facultad de Ingeniería y Ciencias, Universidad Adolfo Ibáñez, Viña del Mar 2580335, Chile; juan.vivanco@uai.cl

**Keywords:** aminochrome, Parkinson’s disease, protein aggregation, ubiquitin, vimentin; autophagy, microtubules

## Abstract

Aminochrome, an endogenous neurotoxin, has been implicated in the loss of neuromelanin-containing dopaminergic neurons in the nigrostriatal system in Parkinson’s disease. Although aminochrome-induced oxidative stress and its inhibitory effects on microtubule polymerization are well documented, its impact on protein aggregation remains poorly understood. The aim of this research was to evaluate the effects of aminochrome on protein aggregate accumulation in SH-SY5Y cells differentiated into dopaminergic neurons. While the role of aminochrome in autophagy has been described, its direct effect on autophagosome–lysosome fusion has not been studied. Our findings reveal that aminochrome, like vinblastine, delays autophagosome–lysosome fusion and induces cell death. This inhibitory effect was also observed in the presence of autophagy inducers, which partially attenuated aminochrome-induced cell death. Under these conditions of disruptions in autophagosome–lysosome fusion, a marked accumulation of perinuclear vimentin and ubiquitin aggregates was observed. Aminochrome also increased colocalization between vimentin and ubiquitin. Interestingly, ubiquitin aggregates were also detected within the nucleus. These findings suggest that aminochrome-induced disruption of the microtubule network, particularly its impairment of autophagosome–lysosome fusion and promotion of protein aggregation, may represent a critical mechanism leading to cell death. In addition, inhibition of autophagosome–lysosome fusion may contribute to the accumulation of perinuclear and nuclear protein aggregates, which may be associated with either toxic or non-toxic pathways. Our findings underscore the therapeutic potential of targeting both microtubule stabilization and proteostasis pathways, including autophagy and the ubiquitin–proteasome system (UPS), in Parkinson’s disease, highlighting the need for further research into nuclear proteotoxicity mechanisms.

## 1. Introduction

Parkinson’s disease (PD) is a neurodegenerative disorder characterized by the progressive degeneration of dopaminergic neurons in the substantia nigra pars compacta (SNpc). A common feature observed in postmortem brains of individuals with PD is the presence of abnormal protein aggregates in soma and neurites of neurons, known as Lewy’s bodies and Lewy’s neurites, respectively [1]. The accumulation of these protein aggregates is recognized as a contributing factor to the neurodegenerative process in both sporadic and hereditary forms of PD [2].

The clearance of protein aggregates is a normal process in neurons, involving the UPS and the autophagy pathway. The latter relies on the formation of autophagosomes and their subsequent fusion with lysosomes for recycling. Aggregated and misfolded proteins are ubiquitinated and recognized by proteaosomes for degradation [3]. Under prolonged stress conditions, the accumulation of these protein aggregates can lead to the formation of perinuclear inclusions known as aggresomes. These structures are composed of aggregated, ubiquitinated proteins and intermediate filaments among other proteins. Vimentin, an intermediate filament, forms a cage surrounding aggregated proteins and is described as a marker of aggresomes [4,5,6]. An altered fusion process between autophagosomes and lysosomes is known to impact the clearance of protein aggregates. Deficient protein clearance mechanisms and the accumulation of protein aggregates are common features of several neurodegenerative disorders, including PD [7].

Autophagy inductors may facilitate the clearance of protein aggregates. For instance, rapamycin and trehalose are compounds that stimulate autophagosome–lysosome fusion and are known to exert neuroprotective effects. Rapamycin functions as an inhibitor of mTOR, thereby altering the PI3K/AKT/mTOR signaling pathway, whereas trehalose can act in both an mTOR-dependent or -independent manner [8,9]. Both compounds modulate autophagy and promote mechanisms for clearing protein aggregates in neurodegenerative processes [10,11,12]. In contrast, bafilomycin A1, a vacuolar-type H+-ATPase inhibitor, prevents the fusion of autophagosomes with lysosomes by either disrupting V-ATPase-mediated acidification or Ca^2+^ depletion via the SERCA pump. It not only provides protection but also induces toxicity in PD models [13,14,15,16,17].

Although protein aggregation is considered an early event in the development of PD, it does not appear to be the initial event. The disruption of the microtubule cytoskeleton network may precede it [18,19,20]. Microtubules, functioning as molecular motors that control vesicle trafficking within cells, are crucial for vesicle fusion, which is necessary for the clearance of protein aggregates. Indeed, studies on models of PD have identified several neurotoxins that disrupt the microtubule network [21,22,23,24]. Similarly, drugs that depolymerize microtubules, such as vinblastine, are known to be neurotoxic [25]. In contrast, drugs that stabilize microtubules network act as neuroprotectors [23]. Nevertheless, the mechanism by which neurotoxins that disrupt the microtubule network impact neuronal degeneration remain to be elucidated.

It has been proposed that aminochrome is the endogenous neurotoxin that triggers neurodegeneration of dopaminergic neurons of the nigrostriatal system under a single-neuron degeneration model [26,27]. The synthesis of neuromelanin, which requires the formation of the neurotoxin aminochrome, is a normal and harmless process due to the presence of DT-diaphorase and glutathione transferase M2-2, which prevent the neurotoxic effects of aminochrome [26]. Aminochrome neurotoxicity is associated with the formation of aggregates of α- and β-tubulin [28,29] and the inhibition of microtubule polymerization through the formation of adducts with tubulin. Interestingly, aminochrome’s effects on the microtubule network are evident prior to cell death [23]. Aminochrome can disrupt microtubule-network-dependent processes, including mitochondrial transport, vesicular trafficking, and autophagy. Although some studies have reported the effects of aminochrome on the clearance mechanisms of misfolded or altered proteins [30], the specific mechanisms by which aminochrome-induced microtubule alterations contribute to neuronal death are not yet fully understood.

The aim of this research was to determine whether aminochrome induces the accumulation of protein aggregates. To address this, we first examined whether aminochrome disrupts autophagosome–lysosome fusion. We then assessed whether aminochrome leads to the accumulation of intracellular protein aggregates.

## 2. Materials and Methods

### 2.1. Reagents

Dopamine and tyrosinase were purchased from Sigma-Aldrich (St. Louis, MO, USA; H8502 and T3824-50KU, respectively). For viability/cytotoxicity analysis, calcein AM (Invitrogen, Thermo Fisher, Rockford, IL, USA; L3224) and propidium iodide (Sigma-Aldrich, St. Louis, MO, USA; P4864) were used. For QRT-PCR analysis, a Brilliant III Ultra Fast SYBR Green QRT-PCR Master Mix from Agilent Technologies (Agilent Technologies, Santa Clara, CA, USA; 600886) was used. For immunofluorescence analysis, the following reagents were used: paraformaldehyde (PFA) (Winkler, Santiago, Chile; 1.04005.1000) and DAKO fluorescent mounting medium (Agilent Technologies, Santa Clara, CA, USA; S3023). The following primary antibodies were used: anti-LAMP-1 (Santa Cruz Biotechnology, Dallas, TX, USA; sc-17768), anti-ubiquitin (Santa Cruz Biotechnology, Dallas, TX, USA; sc-271289) and anti-vimentin (Santa Cruz Biotechnology, Dallas, TX, USA; sc-5565), from Santa Cruz Biotechnology; and anti-LC3β (Abcam, Cambridge, MA, USA; ab51520) and anti-vimentin (Abcam, Cambridge, MA, USA; ab8978), from Abcam. The following secondary antibodies were also used: Alexa Fluor 488 donkey anti-rabbit (Jackson Immunoresearch, West Grove, PA, USA; 711-545-152), Alexa Fluor 488 goat anti-mouse IgG (Jackson Immunoresearch, West Grove, PA, USA; 115-545-003) and Cy3™ Goat anti-mouse IgG (Jackson Immunoresearch, West Grove, PA, USA; 115-165-166) from Jackson Immunoresearch, and Alexa Fluor 546 goat anti-rabbit (Thermo Fisher Scientific, Rockford, IL, USA; A11035) and Alexa Fluor 546 goat anti-mouse (Thermo Fisher Scientific, Rockford, IL, USA; A11030) from Thermo Fisher Scientific. To label the nucleus, Hoechst (Thermo Fisher Scientific, Rockford, IL, USA; H3569) was used. For the differentiation procedure, retinoic acid (Santa Cruz Biotechnology, Dallas, TX, USA; sc-200898) and TPA (12-O-tetradecanoylphorbol-13-acetate; Santa Cruz Biotechnology, Dallas, TX, USA; sc-202021) from Santa Cruz Biotechnology were used. For cell treatment, vinblastine acquired from Laboratorio Chile under the name of Lemblastine (Laboratorio Chile, Santiago, Chile), trehalose (Sigma-Aldrich, St. Louis, MO, USA; T0167) from Sigma-Aldrich, and rapamycin (Santa Cruz Biotechnology, Dallas, TX, USA; sc-3504A) and bafilomycin A1 (Santa Cruz Biotechnology, Dallas, TX, USA; sc-201550A), both purchased from Santa Cruz Biotechnology, were used.

### 2.2. Cell Culture and Differentiation

The cell culture and differentiation protocol were followed as described in Briceño [23]. The SH-SY5Y cell line was purchased from ATCC (ATCC, Manassas, VA, USA; CRL-2266). The growth medium used was DMEM/F12 (Dulbecco’s Modified Eagle medium/F12 1:1) (Hyclone, Logan, UT, USA; sh30004.04) supplemented with 7.5% adult bovine serum (Sartorius, Biological industries, Kibbutz Beit Haemek, Israel; 04-003-1B), 1.5% fetal bovine serum (Sartorius, Biological industries, Kibbutz Beit Haemek, Israel; 04-001-1A), 1X of non-essential amino acid solution (MEM NEAA 100X) (Hyclone, Logan, UT, USA; sh30238.01), and 2X penicillin–streptomycin–amphotericin B solution (Sartorius, Biological industries, Kibbutz Beit Haemek, Israel; 03-033-1B) at a concentration of 200 U/mL of penicillin G sodium salt, 0.2 mg/mL of streptomycin sulfate, and 0.5 µg/mL of amphotericin B [31]. Cell cultures were kept in an incubator at 37 °C with an atmosphere of 5% CO_2_. When cells reached at 80% confluence, the differentiation of SH-SY5Y cells into dopaminergic neurons was initiated. Differentiation was carried out in two steps: In the first step, the cells were cultured for 3 days in DMEM/F12 media (supplemented with 4% bovine serum, 1X MEM, and 2X penicillin–streptomycin–amphotericin B solution) containing 10 μM retinoic acid. In the second stage, the cells were cultured for 3 more days in the same media but instead of retinoic acid, 80 nM TPA (12-O-tetradecanoylphorbol-13-acetate) was used [32,33]. After 6 days of differentiation, the treatments were carried out for 0.5, 4, 9, 14 or 16 h with 50 μM aminochrome. Also, the cells were incubated with 10 μM rapamycin, 5 nM bafilomycin A1, 100 mM trehalose or 10 μM vinblastine in the presence or absence of 50 μM aminochrome for 16 h. Cells were treated with aminochrome in serum-free media for 1.5 h. After this time, the media was removed and replaced with fresh growth media, maintaining the other treatments until incubation time was completed. For treatments with bafilomycin A1 (5 nM), rapamycin (10 µM), or trehalose (100 mM), cells were preincubated for 24 h.

### 2.3. Synthesis and Purification of Aminochrome

The synthesis and purification protocol for aminochrome was followed as described by Briceño et al. [23]. Dopamine (7.5 mmol) and 10 ng of tyrosinase were incubated in 25 mM potassium phosphate buffer at pH 6 for 15–20 min at room temperature. To purify aminochrome, the incubation solution was loaded onto a CM-Sephadex C50-1000 (18 × 0.7 cm) column (Sigma-Aldrich, C25120). The red-orange solution corresponding to aminochrome was collected and detected spectrophotometrically by measuring the absorbance at 480 nm. Aminochrome concentration was determined by the molar extinction coefficient of 3058 M^−1^ cm^−1^ [34].

### 2.4. Assessment of Cell Viability and Death

The protocol for assessing cell death was followed as described in Briceno et al. [23]. For live/dead experiments, cells were cultured in 24-well dishes according to a previously described culture and differentiation protocol. Cell death was determined by using calcein AM and propidium iodide, two fluorescent reagents that discriminate the population of live cells from the dead cell population. After aminochrome treatment for 16 h at 37 °C, SH-SY5Y cells were washed with PBS (Dulbecco’s Phosphate-Buffered Saline) (Biological Industries, 02-023-5A) 3 times for 5 min. Then, cells were incubated for 30 min in the dark with 1.5 μM of calcein AM and 1 μM of propidium iodide, both prepared in PBS. Live and dead cell counting was performed using a Leica fluorescence microscope DM1L (Leica, Wetzlar, Germany). One hundred cells per replicate were counted.

### 2.5. Immunofluorescence LC3β-LAMP1 Double Staining

SH-SY5Y cells were cultured and differentiated on 12 mm gelatin-coated coverslips placed in 24-well plates. Cells were treated with 50 µM aminochrome for 16 h at 37 °C, washed with PBS, and fixed with 4% paraformaldehyde (PFA) for 30 min. To permeabilize the membrane and facilitate LC3β-I exposure, cells were incubated with 0.5% saponin for 15 min in the dark. Blocking was performed for 1 h using 6% bovine serum albumin (BSA). For LC3β and LAMP1 colocalization studies, cells were incubated separately overnight at 4 °C with rabbit anti-LC3β antibody (1:2000) and mouse anti-LAMP1 antibody (1:100). Secondary antibodies, Alexa Fluor 488 donkey anti-rabbit and Alexa Fluor 546 goat anti-mouse (both at 1:200 dilution), were applied separately for 1.5 h. Nuclear staining was performed with Hoechst reagent (1:10,000) for 5 min. Coverslips were mounted on slides using DAKO fluorescent mounting medium. Images were acquired using a Carl Zeiss Observer.Z1 Axio fluorescence microscope (Göttingen, Germany) and analyzed with AxioVision Rel 4.8 software for deconvolution, colocalization and quantification of the mean fluorescence intensity.

### 2.6. Immunofluorescence Vimentin Staining

SH-SY5Y cells were cultured and differentiated in 24 well plates on 12 mm coverslips previously gelatinized. Treatments were carried out in the presence and absence of 50 μM aminochrome for 16 h at 37 °C. Cells were washed with 1X Tris-buffered saline (TBS) and fixed with 4% PFA for 30 min. The cells were blocked for 1 h with 6% BSA and 0.5% Triton X-100 in 1X TBS. The cells were incubated with a primary antibody 1:100 dilution of rabbit anti-vimentin overnight at 4 °C. After washing the cells with TBS, a 1:200 dilution of secondary antibody (AF-546 goat anti-rabbit IgG) was incubated for 1.5 h. For nucleus staining, Hoechst reagent was used at a 1:10,000 dilution in Tris–EDTA buffer for 5 min. Finally, coverslips were mounted onto slides using DAKO fluorescent mounting medium. Images were acquired using a Carl Zeiss Observer.Z1 Axio fluorescence microscope (Göttingen, Germany) and analyzed with AxioVision Rel 4.8 software for deconvolution.

### 2.7. Immunofluorescence Vimentin-Ubiquitin Double Staining

SH-SY5Y cells were cultured and differentiated on 12 mm gelatin-coated coverslips in 24-well plates. Treatments with 50 μM aminochrome were performed for 16 h at 37 °C, with untreated controls included. Cells were washed with 1X TBS and fixed with 4% PFA for 30 min. Blocking was carried out for 1 h in 6% bovine serum albumin (BSA) and 0.5% Triton X-100 in 1X TBS. Cells were incubated overnight at 4 °C with mouse anti-ubiquitin primary antibody (1:250 dilution) in 6% BSA and 0.5% Triton X-100 in 1X TBS. After washing, cells were incubated for 1.5 h in the dark with Alexa Fluor 488 goat anti-mouse IgG secondary antibody (1:200 dilution) in 3% BSA in 1X TBS. Subsequently, cells were incubated overnight at 4 °C in the dark with mouse anti-vimentin primary antibody (1:1000 dilution) in 6% BSA and 0.5% Triton X-100 in 1X TBS. Following washes, cells were incubated for 1.5 h in the dark with Cy3™ goat anti-mouse IgG secondary antibody (1:800 dilution) in 3% BSA in 1X TBS. Nuclear staining was performed using Hoechst reagent (1:10,000 dilution) in Tris–EDTA buffer for 5 min. Coverslips were mounted on slides using Dako fluorescent mounting medium and stored at 4 °C in the dark. Images were acquired using a Carl Zeiss Observer.Z1 Axio fluorescence microscope (Göttingen, Germany) and analyzed with AxioVision Rel 4.8 software for deconvolution, including extended focus.

### 2.8. Quantitative Real-Time PCR

The total RNA from SH-SY5Y cells was extracted with TRIZOL reagent (Invitrogen, 15596-026) according to the manufacturer’s protocol and was quantified using a Nanodrop 3300 (Thermo Fisher Scientific, Rockford, IL, USA). The cDNA was synthesized using oligo-dT (IDT) and Epicenter RT reagents according to the manufacturer’s instructions. Comparative quantitative real-time PCR for the *UBI* gene (Fw: 5′-CGCACCCTGTCTGACTACAA-3′; Rv: 5′AGGGATGCCTTCCTTGTCTT-3′), *LC3* gene (Fw: 5′-CAGTACTTGCATGGGGTTCA-3′; Rv: 5′-TCTGGTTTTCCCCGTTACAG-3′) and *LAMP1* gene (Fw: 5′-CATCAACCCCAACAAGACCT-3’; Rv: 5′-TCTCTGGCTTCAGGAAGAAT-3′) was performed using Brilliant III Ultra Fast SYBR Green QRT-PCR Master Mix in a Stratagene Mx3000p Detection system (Agilent Technologies, Santa Clara, CA, USA). The mRNA levels were normalized through the housekeeping gene *GAPDH* (Fw: 5′-GCCAAAAGGGTCATCATCTC-3′; Rv: 5′-TGTGGTCATGAGTCCTTCCA-3′). The following experimental protocol was used: 25 °C for 1 s, 50 °C for 10 min, and 95 °C for 3 min, followed by 40 cycles of 95 °C for 20 s, 60 °C for 20 s and a last segment that includes 95 °C for 1 min, 55 °C for 30 s and 95 °C for 30 s. v4.0 MxPro Software was used to analyze real-time data.

### 2.9. Statistical Analysis

All data were expressed as mean ± SEM. Statistical significance was assessed using analysis of variance (ANOVA) for multiple comparisons followed by the Newman–Keuls post hoc test, while an unpaired *t*-test was used for two-group comparisons. In all cases, the significance level was set to α = 0.05. Normality was assessed using the Kolmogorov–Smirnov test.

## 3. Results

### 3.1. Aminochrome and Vinblastine Inhibit the Fusion of Autophagosome with Lysosomes in the Presence of Autophagy Inducers in SH-SY5Y Cells

**Figure 1:**
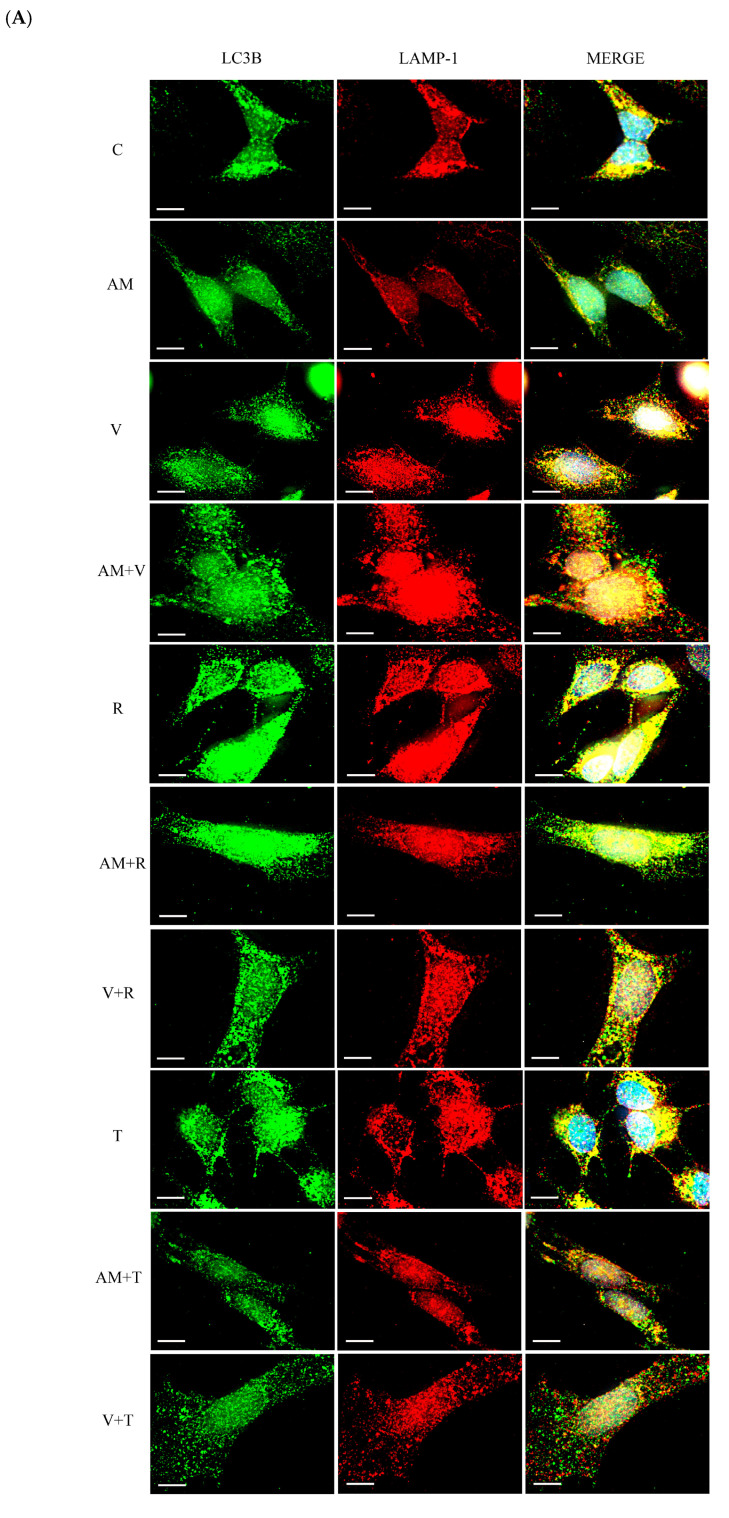

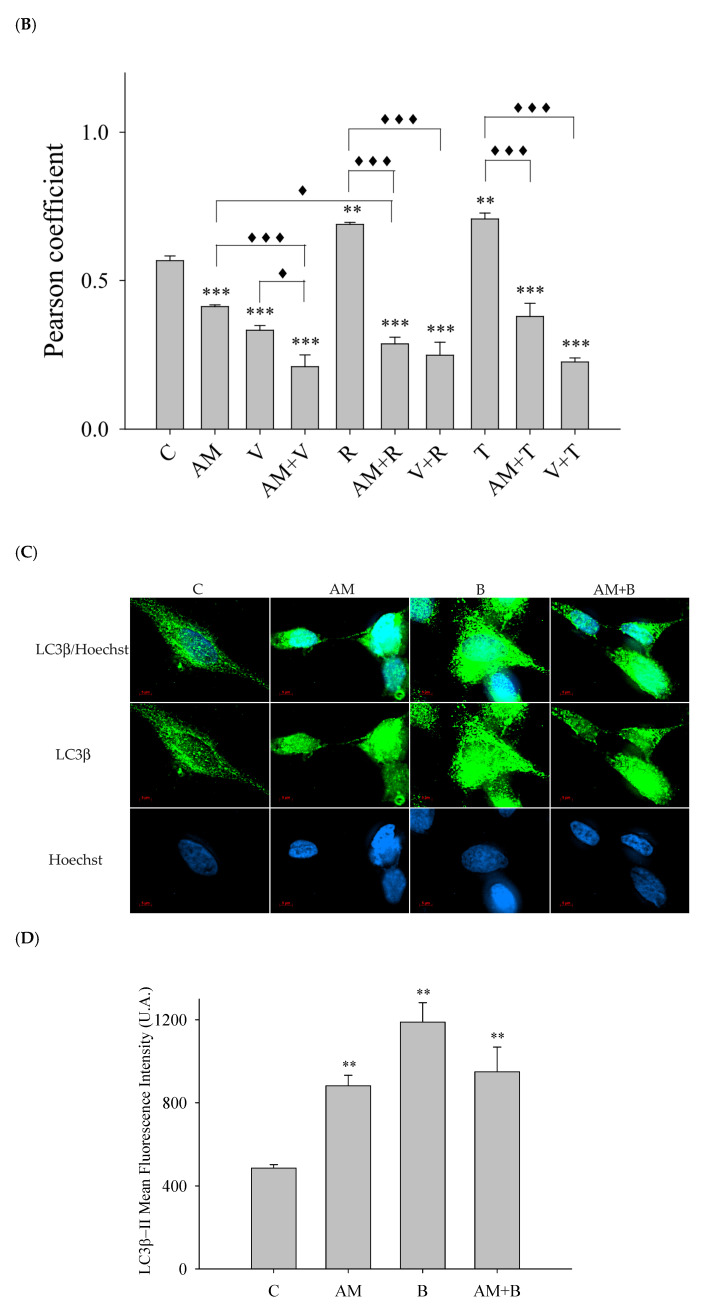

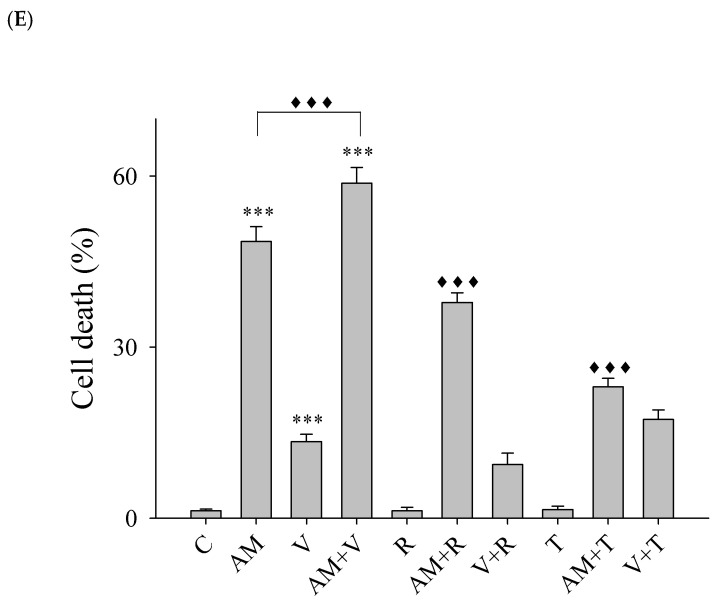
Effect of aminochrome and autophagy inducers on autophagosome–lysosome fusion in SH-SY5Y cells. (**A**) Immunofluorescence double labeling of microtubule-associated protein light chain (LC3β) (green) and lysosomal-associated membrane protein 1 (LAMP1) (red) in SH-SY5Y cells treated with 50 µM aminochrome (AM), 10 µM vinblastine (V), 50 µM aminochrome and 10 µM vinblastine (AM + V), 10 µM rapamycin (R), 50 µM aminochrome and 10 µM rapamycin (AM + R), 10 µM vinblastine and 10 µM rapamycin (V + R), 100 mM trehalose (T), 50 µM aminochrome and 100 mM trehalose (AM + T), or 10 µM vinblastine and 100 mM trehalose (V + T) for 16 h at 37 °C. As a control, the cells were incubated only in culture medium (C). Scale bar: 10 µm. (**B**) Quantification of the Pearson coefficient based on immunofluorescence double labeling shown in (**A**). (**C**) Immunofluorescence of microtubule-associated protein light chain (LC3β) (green) in SH-SY5Y cells, which were treated with 50 µM aminochrome (AM), 5 nM bafilomycin (B), or 50 µM aminochrome plus 5 nM bafilomycin (AM + B) for 16 h at 37 °C. As a control, the cells were incubated only in culture medium (C). (**D**) Quantification of LC3β -II mean fluorescence intensity in saponinated cells based on LC3β labeling shown in (**C**). (**E**) Quantification of cell death. The images were obtained with a fluorescence microscope (Carl Zeiss, Göttingen, Germany; Observer.Z1 Axio model) and analyzed with the AxioVision Rel 4.8 software for deconvolution, colocalization and quantification of the mean fluorescence intensity. The statistical significance was assessed using analysis of variance (ANOVA) for multiple comparisons and the Newman–Keuls test (** *p* < 0.01 and *** *p* < 0.001 compared with control; ♦ *p* < 0.05 and ♦♦♦ *p* < 0.001 compared among experimental groups differing from control). The experiment was performed with three independent replicates. Additional data supporting these findings are provided in Supplementary Tables S1–S3.

In order to determine whether aminochrome affects the fusion of autophagosome with lysosomes in SH-SY5Y cells, immunofluorescence double staining for microtubule-associated proteins light chain 3β (LC3β) and lysosomal-associated membrane protein 1 (LAMP1) (Figure 1A) was performed. Colocalization analysis was conducted, and the Pearson correlation coefficient was quantified (Figure 1B) [35]. A significant decrease in the Pearson coefficient was observed in the presence of 50 µM aminochrome compared with the control without aminochrome (27% decrease, *p* ˂ 0.001). Subsequently, we evaluated whether alterations in the microtubule network are primarily responsible for the inhibition of autophagosome–lysosome fusion in SH-SY5Y cells, as well as the role of autophagy inducers. The cells were incubated with aminochrome in the presence of vinblastine, a microtubule-depolymerizing agent [36,37], and the autophagy inducers rapamycin and trehalose. A further significant decrease in the Pearson coefficient (63%, *p* < 0.001) was observed in the presence of 50 µM aminochrome and vinblastine compared with aminochrome. Moreover, treatment with rapamycin, an autophagy inducer, resulted in a significant increase (22%, *p* ˂ 0.01) in the Pearson coefficient compared with control conditions; however, this effect was markedly reduced by aminochrome and vinblastine (58% and 64%, *p* ˂ 0.001), respectively. The aminochrome-induced decrease in the Pearson coefficient was further enhanced when autophagy was previously induced with rapamycin, but not with trehalose (30%, *p* < 0.05), compared with aminochrome alone. Similarly, treatment with trehalose, another autophagy inducer [38], increased the Pearson coefficient (25%, *p* < 0.01) compared with control conditions; however, this effect was also significantly inhibited by aminochrome and vinblastine (46% and 68% decrease; *p* < 0.001) respectively. To assess whether aminochrome-induced inhibition of autophagosome–lysosome fusion leads to LC3β-II accumulation, mean LC3β fluorescence intensity was quantified from immunofluorescence images in saponin-treated cells. Aminochrome, similar to the lysosomal inhibitor bafilomycin A1, significantly increased LC3β-II levels compared to the control (81% and 145%, respectively; *p* < 0.01). A comparable increase was observed in cells treated with aminochrome plus bafilomycin A1 (95%, *p* < 0.01) (Figure 1D). Although aminochrome significantly increased LC3β-II levels, no additional increase was observed upon co-treatment with bafilomycin A1 compared with aminochrome alone. The lack of further LC3β-II accumulation in the presence of bafilomycin A1 suggests that aminochrome impairs autophagic flux. These results suggest that microtubules-depolymerizing agents, such as aminochrome and vinblastine, impair autophagosome–lysosome fusion even in the presence of autophagy inducers in SH-SY5Y cells.

### 3.2. Aminochrome Toxicity Is Enhanced in the Presence of Vinblastine but Inhibited by Autophagy Inductors

To evaluate whether microtubule disruption is a primary event responsible for cell death through inhibition of autophagosome–lysosome fusion in SH-SY5Y cells, cell death induced by aminochrome was assessed in the presence of vinblastine, a well-known microtubule-depolymerizing agent, using calcein AM and propidium iodide. In parallel, cell death was also evaluated in the presence or absence of the autophagy inducers rapamycin and trehalose (Figure 1E). A significant increase in the cell death (37-fold, *p* ˂ 0.001) was observed in the presence of 50 µM aminochrome compared with control conditions, which was further enhanced (21%, where *p* ˂ 0.001) in the presence of vinblastine. Moreover, treatment with rapamycin in the presence of aminochrome significantly reduced cell death (22%, *p* < 0.001) compared with aminochrome alone. Similarly, trehalose treatment also decreased aminochrome-induced cell death (53%, *p* < 0.001). Additionally, a significant increase in cell death (10-fold, *p* ˂ 0.001) was observed in the presence of vinblastine compared with the control. In contrast, co-treatment with trehalose or rapamycin did not significantly alter vinblastine-induced cell death (Figure 1E). Consequently, aminochrome-induced toxicity is exacerbated by microtubule depolymerization but partially attenuated by autophagy induction, despite the increased inhibition of autophagosome–lysosome fusion under these conditions.

### 3.3. Aminochrome-Induced Increase in the Formation of Perinuclear Vimentin Aggregates and Nuclear Ubiquitin Aggregates in SH-SY5Y Cells

**Figure 2:**
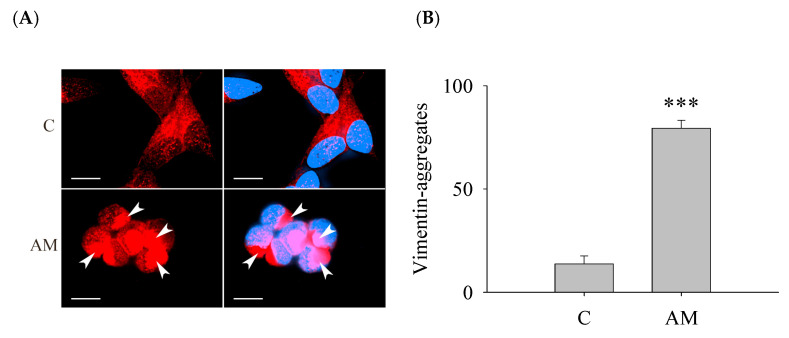
Vimentin aggregation induced by aminochrome in SH-SY5Y cells. (**A**) Immunofluorescence labeling of vimentin (red) in SH-SY5Y cells treated with 50 µM aminochrome (AM), for 16 h at 37 °C. As a control, the cells were incubated only in culture medium (C). Scale bar: 10 µm. (**B**) Quantification of vimentin aggregates. The images were obtained with a fluorescence microscope (Carl Zeiss, Göttingen, Germany; Observer.Z1 Axio model) and analyzed for deconvolution with the AxioVision Rel 4.8 software. The statistical significance was assessed using unpaired *t*-tests for two-group comparisons (*** *p* < 0.001 compared with control). The experiments were performed with four independent replicates. Additional data supporting these findings are provided in Supplementary Table S4.

When the cellular protein-folding capacity is exceeded, misfolded and aggregated proteins are primarily eliminated through two major quality-control systems: the UPS and autophagy. These pathways are responsible for eliminating damaged organelles and protein complexes, including aggregates of misfolded proteins known as aggresomes. These structures generally arise when the burden of misfolded proteins exceeds the degradative capacity of the UPS [3]. Aggresomes are typically organized within a cage-like network of vimentin intermediate filaments and are subsequently removed through selective autophagy [4,5,6,39]. In this study, it was observed that alterations in the microtubule network induced by aminochrome are a critical event affecting autophagosome–lysosome fusion, leading to cell death. Our objective was to investigate whether, under these conditions, aminochrome also induces the formation of perinuclear vimentin aggregates. Immunofluorescence staining for vimentin (Figure 2A) was performed, and the quantification of vimentin aggregates in the presence of aminochrome was conducted (Figure 2B). A significant increase (6-fold, *p* ˂ 0.001) in perinuclear vimentin aggregates was observed in the presence of 50 µM aminochrome compared with the control without aminochrome (Arrowhead, Figure 2A).

**Figure 3:**
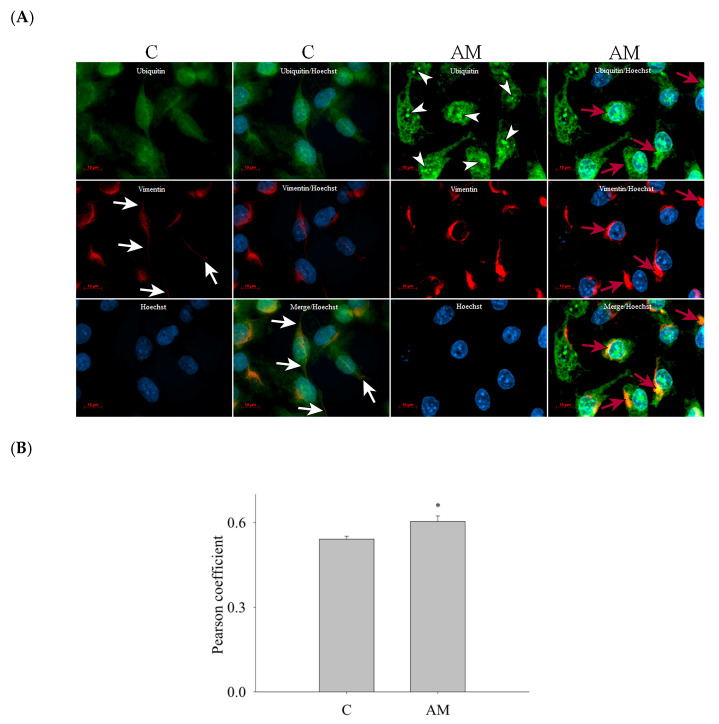

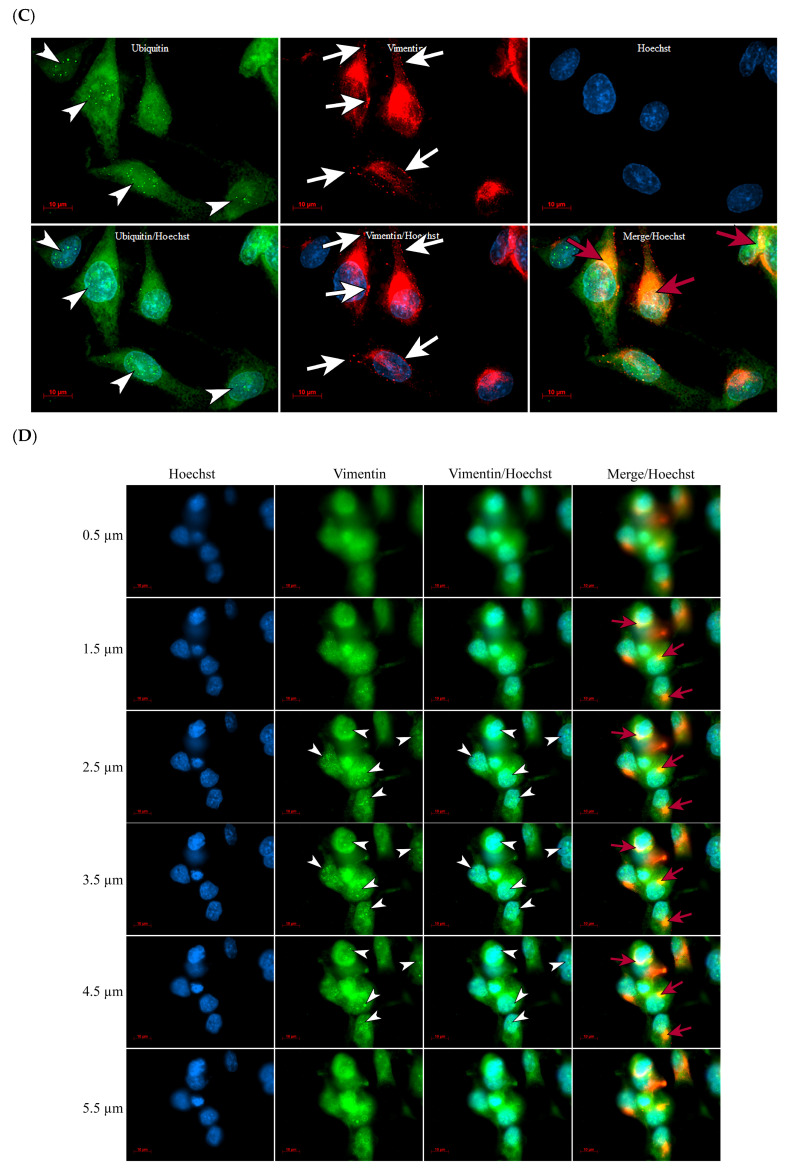
Aminochrome-induced vimentin and nuclear ubiquitin aggregation in SH-SY5Y cells. (**A**) Immunofluorescence double labeling of ubiquitin (green) and vimentin (red) in SH-SY5Y cells treated with 50 µM aminochrome (AM) for 16 h at 37 °C. As a control, the cells were incubated only in culture medium (C). (**B**) Quantification of the Pearson coefficient based on immunofluorescence double labeling shown in (**A**). (**C**) Immunofluorescence images showing vimentin aggregations at proximal and distal sites relative to the perinuclear region. (**D**) Z-stack images (0.5 to 5.5 µm) of immunofluorescence double labeling for ubiquitin (green) and vimentin (red), highlighting ubiquitin aggregates in the nuclear region. The images were obtained with a fluorescence microscope (Carl Zeiss, Göttingen, Germany; Observer.Z1 Axio model) and analyzed for deconvolution, colocalization and extended focus with the AxioVision Rel 4.8 software. The statistical significance was assessed using unpaired *t*-tests for two-group comparisons (* *p* < 0.05 compared with control). The experiments were performed in three independent replicates. Additional data supporting these findings are provided in Supplementary Table S5.

Subsequently, we investigated the effect of aminochrome on the cellular distribution and colocalization of ubiquitin and vimentin. Immunofluorescence double staining for vimentin and ubiquitin was performed (Figure 3A,C,D), and image analyses for colocalization and quantification of the Pearson coefficient were conducted (Figure 3B). An extended-focus analysis of the deconvolved images in Z-stack was provided. Similar to the results observed in Figure 2A, a homogeneous cytoplasmic vimentin labeling was noted (white arrow), which changed to a perinuclear cellular distribution in the presence of aminochrome compared with the control (red arrow). In the case of ubiquitin staining, it appeared in the cytoplasm and nucleus but was mainly concentrated in perinuclear (red arrow) and nuclear (arrowhead) regions in the presence of aminochrome compared with the control (Figure 3A). Ubiquitin in the perinuclear region colocalized with vimentin (red arrow, Figure 3A), where a significant increase (12%, *p* ˂ 0.05) in the Pearson coefficient was observed in the presence of 50 µM aminochrome compared with the control (Figure 3B). These aggregations were observed not only in perinuclear regions but also in regions proximal or distal to the perinuclear region within cellular processes when the cells were treated with aminochrome (white arrow, Figure 3C). This image also highlights nuclear ubiquitin labeling (arrowhead) and vimentin–ubiquitin colocalization (red arrow) (Figure 3C). Z-stack images from 0.5 to 5.5 µm demonstrate the correspondence between ubiquitin labeling and nuclear regions (arrowhead). Likewise, ubiquitin colocalizes with vimentin within the perinuclear space (red arrow) (Figure 3D). These results demonstrate that aminochrome induces the formation of cellular processes/perinuclear vimentin aggregates and nuclear ubiquitin aggregates in SH-SY5Y cells.

### 3.4. Aminochrome-Induced Increase in LC3β and Ubiquitin Expression in SH-SY5Y Cells

**Figure 4:**
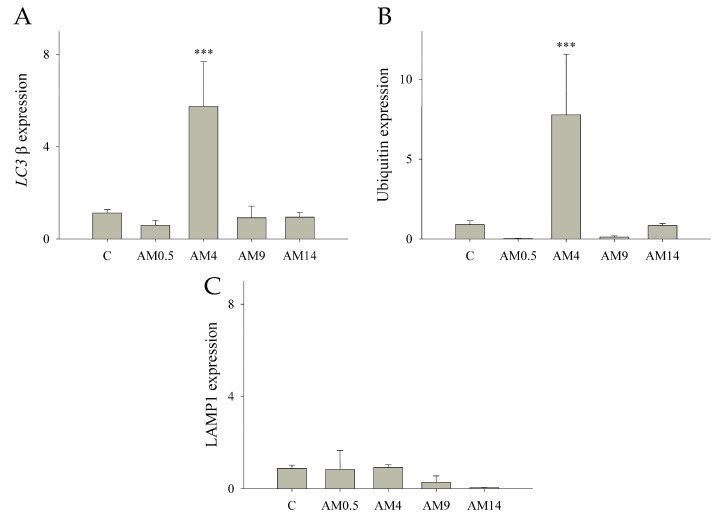
*LC3β*, ubiquitin and LAMP1 expression induced by aminochrome in SH-SY5Y cells. Quantitative real-time PCR analysis. Total RNA from SH-SY5Y cells treated with 50 µM aminochrome for 0.5, 4, 9 and 14 h at 37 °C and 5% CO_2_ was extracted as described under experimental procedures. mRNA levels were normalized using the housekeeping gene *GAPDH*. (**A**) *LC3β* mRNA expression. (**B**) Ubiquitin mRNA expression. (**C**) LAMP1 mRNA expression. Statistical significance was assessed using analysis of variance (ANOVA) for multiple comparisons and the Newman–Keuls test (*** *p* < 0.001 compared with control). The experiment was performed in three to six independent replicates. Additional data supporting these findings are provided in Supplementary Tables S6–S8.

In this study, it was observed that aminochrome affects autophagosome–lysosome fusion and alters the clearance of protein aggregates. Our objective was to investigate whether aminochrome also induces changes in the expression of genes related to autophagy and the UPS, such as LC3β, LAMP1 and ubiquitin. Previously, it was reported that aminochrome affects TUBB3 expression, with this effect possibly being a compensatory mechanism to protect against cellular damage [23]. In this context, it was investigated whether early changes in the expression of LC3β, LAMP1 and ubiquitin were observed. Gene expression was measured using qRT-PCR. It was observed that when cells were incubated in the presence of 50 µM aminochrome, a significant increase (fivefold, *p* ˂ 0.001) in LC3β gene expression at 4 h compared with the control was noted (Figure 4A). Similarly, a significant increase (ninefold, *p* ˂ 0.001) in ubiquitin expression at 4 h compared to the control was observed (Figure 4B). No significant changes in LAMP1 gene expression were observed (Figure 4C).

**Figure 5:**
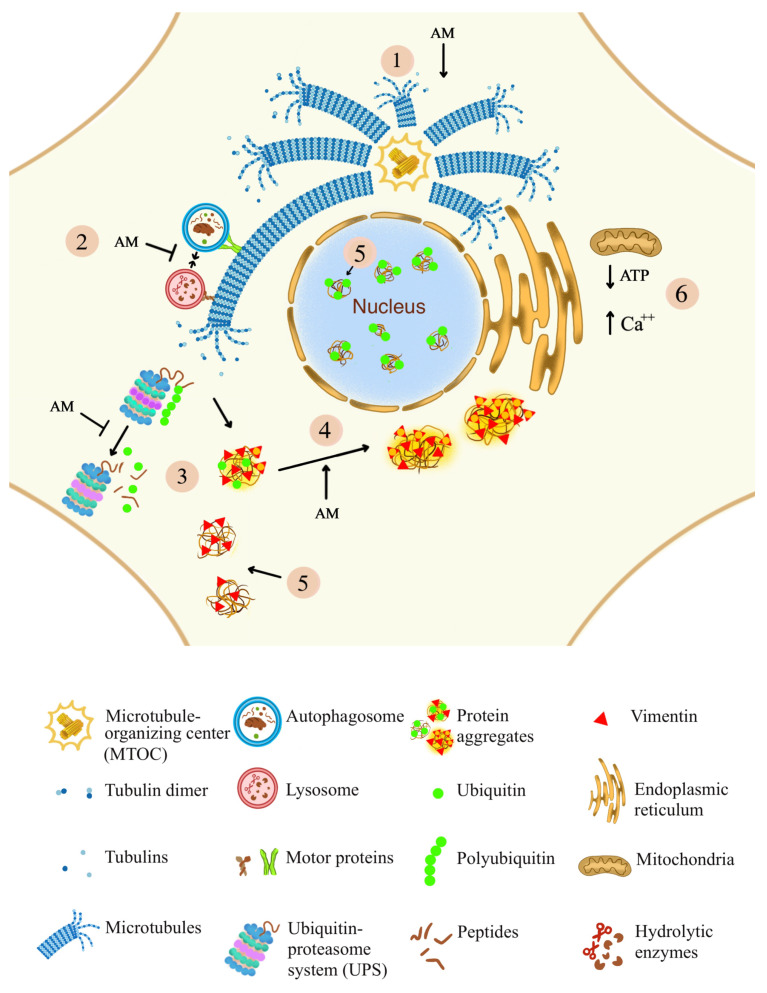
Possible mechanism of action of aminochrome on autophagosome–lysosome fusion and protein aggregate formation. Aminochrome forms adducts with tubulin and induces microtubule depolymerization (**1**), altering the fusion of autophagosomes with lysosomes (**2**). Decreased fusion between autophagosomes and lysosomes and disrupted UPS could favor the accumulation of protein aggregates (**3**). Aminochrome promotes the formation of vimentin aggregates and ubiquitin aggregates toward perinuclear regions that colocalize with ubiquitin (**4**). Aminochrome promotes vimentin aggregation within cellular processes, concomitant with the accumulation of nuclear ubiquitin aggregates (**5**). However, the initial effects of aminochrome on the microtubule network and possibly on autophagy and UPS mechanisms are not the only ones that could explain its toxic effects. A series of events could be triggered directly or indirectly by aminochrome, in which dysregulation of calcium signaling and mitochondrial dysfunction may play significant roles, and in which protein aggregate formation may represent either toxic or adaptive responses (**6**).

A possible mechanism by which aminochrome affects autophagosome–lysosome fusion and promotes protein aggregates formation is illustrated in the following figure (Figure 5).

## 4. Discussion

### 4.1. Aminochrome-Induced Disruption of Autophagosome-Lysosome Fusion

In this research we evaluate the effects of aminochrome on protein aggregate accumulation in SH-SY5Y cells differentiated into dopaminergic neurons. While the role of aminochrome in autophagy has been described, its direct effect on autophagosome–lysosome fusion has not been studied. Our findings reveal that aminochrome toxicity is mediated, in part, by inhibition of autophagosome–lysosome fusion, an effect that is potentiated by vinblastine, a microtubule-depolymerizing agent. Several mechanisms could underlie this fusion impairment, including microtubule instability and dysregulation of cytosolic calcium levels [15]. We previously reported that aminochrome inhibits microtubule polymerization via the formation of adducts with tubulin, reflecting microtubule instability [23]. The fact that vinblastine enhances this inhibition and simultaneously increases cell death suggests that specifically microtubule destabilization may be a key event triggering neuronal death. Microtubule instability impairs autophagosome–lysosome fusion. For instance, vinblastine has been shown to reduce this fusion in HeLa cells and to promote cell death in RCSN-3 cells [25,37]. In addition, agents that stabilize the microtubule network partially reduce aminochrome-induced cell death [23], highlighting the importance of cytoskeletal integrity in this process. Although it remains unclear whether aminochrome has a greater affinity for tubulin than vinblastine, the observed additive effects of aminochrome and vinblastine suggest that aminochrome may not fully saturate microtubule depolymerization. Alternatively, aminochrome and vinblastine may act through distinct but convergent pathways that ultimately impair the fusion of autophagosomes with lysosomes. Aminochrome, like vinblastine, has been shown not only to induce microtubule disassembly but also to cause ATP depletion [40,41].

This inhibitory effect on autophagosome–lysosome fusion was also observed in the presence of autophagy inducers which partially attenuated aminochrome-induced cell death. The data presented here indicate that rapamycin significantly reduces aminochrome-induced neuronal death, despite impairing autophagosome–lysosome fusion. Rapamycin alone increases fusion, suggesting that autophagy is activated under these conditions. Nevertheless, it remains unclear how rapamycin exerts its neuroprotective effect against aminochrome-induced toxicity. Possibly, (i) previous activation of autophagy by rapamycin may facilitate the early clearance of aminochrome-induced protein aggregates, thereby preventing their accumulation and the subsequent engagement of alternative toxic pathways; (ii) another possibility is that, although rapamycin activates autophagy, aminochrome-induced alterations in the microtubule network may impair autophagosome–lysosome fusion, leading to the accumulation of protein aggregates in non-toxic pathways and thereby reducing cytotoxicity.; and (iii) although mTOR inhibition by rapamycin has been associated with neuroprotective effects [42], other studies have shown that inhibition of mTORC1 impairs the structure and function of dopaminergic neurons [43]. In contrast, mTOR activation has been reported to exert neuroprotective effects in degenerating dopaminergic neurons [44]. Based on these observations, the neuroprotective effect of rapamycin against aminochrome toxicity may involve mechanisms independent of mTOR inhibition. Recent studies suggest that rapamycin may exert neuroprotective effects through inhibition of FKBP12 rather than mTORC1 [45]. This could partially explain the observed decrease in autophagosome–lysosome fusion despite the protective effect.

On the other hand, trehalose significantly reduces aminochrome-induced neuronal death, exhibiting a stronger protective effect than rapamycin, without increasing autophagosome–lysosome fusion. Notably, trehalose, similar to rapamycin, increases fusion under basal conditions, suggesting that autophagy is activated under these conditions. Possibly, (i) trehalose may also promote the early clearance of aminochrome-induced protein aggregates, thereby limiting their accumulation and attenuating downstream toxic effects. (ii) The fact that protection of trehalose against aminochrome toxicity is not associated with an increase in fusion suggests that additional mechanisms may contribute to this effect. Although the precise mechanisms underlying trehalose-induced neuroprotection remain unclear, its effects likely involve multiple pathways, including autophagy activation, inhibition of protein aggregation, anti-inflammatory responses, and modulation of JNK/p38 MAPK/AMPK signaling pathways [12,46,47,48]. In summary, inhibition of autophagosome–lysosome fusion may contribute to both toxic and non-toxic cellular states, where protein aggregation may emerge as a possible response to impaired proteostasis.

### 4.2. Aminochrome Induces Nuclear and Perinuclear Aggregated-Protein Formation

Neurons are particularly vulnerable to oxidative damage, which promotes protein oxidation favoring misfolding and abnormal association [49]. Previous studies demonstrated that aminochrome promotes the formation of perinuclear actin and tubulin aggregates, generates adducts with tubulin and destabilizes the microtubule network [23]. In the present study, accumulation of perinuclear vimentin aggregates and cellular projections was induced by aminochrome. Furthermore, both perinuclear and nuclear ubiquitin labeling were detected in the presence of aminochrome. These findings suggest that aminochrome-induced microtubule destabilization and inhibition of autophagosome–lysosome fusion may contribute to protein aggregate accumulation. Consistent with previous reports showing that aminochrome toxicity involves the formation of synuclein oligomers [50] and that misfolded α-synuclein impairs UPS function [51], UPS inhibition may also occur under these conditions. Although the UPS was not directly evaluated in this study, previous evidence indicates that aminochrome disrupts the ubiquitin–proteasome pathway [52], thereby promoting aggresome formation [53]. Here, the results showed that aminochrome increased ubiquitin–vimentin colocalization which could indicate the accumulation of abnormal proteins that are not efficiently eliminated by UPS. While the perinuclear vimentin staining pattern supports the formation of aggresome-like structures, further studies are needed to discriminate whether misfolded protein inclusion bodies and insoluble protein deposits are also generated.

Interestingly, the proportion of cells displaying aggregates is higher than that of dead cells under the same conditions, suggesting that not all cells harboring aggregates are committed to cell death. In fact, we previously reported that the protective effect of rapamycin against aminochrome toxicity was observed under conditions that increase the inhibition of autophagosome–lysosome fusion, suggesting, at least in part, that the accumulation of protein aggregates may not necessarily lead to toxic effects in some cells. In Parkinson’s disease, surviving neurons frequently contain cytoplasmic inclusions known as Lewy bodies [54,55], suggesting that aggregate formation may represent a protective adaptation or an intermediate stage preceding cell death. Additional investigations are required to clarify this relationship. Similarly, it is known that aggresomes require the formation of autophagosomes for their elimination by autophagy [56]. Poly-ubiquitination of misfolded proteins favors both the formation of aggresomes and subsequent elimination by autophagy [57,58]. Since aminochrome toxicity also impairs autophagic–lysosomal dysfunction [29,50], this could further contribute to the accumulation of these aggregates. Indeed, the accumulation of ubiquitinylated protein aggregates in the post-mortem brain of patients with PD suggests a dysfunction of the aggresome–autophagy pathway [57,59]. Possibly, the simultaneous action of several of these mechanisms may act synergistically to promote the formation and accumulation of protein aggregates and, consequently, cell death, where destabilized microtubules determine the initiation of several irreversible processes.

Ubiquitin participates in the clearance of nuclear aggregates through the nuclear proteasomal system [60]. Under conditions of prolonged cellular stress, the formation of nucleolar aggresomes has also been documented [61,62]. The study shows the presence of aminochrome-induced nuclear ubiquitin aggregates, providing new insights into the nuclear effects of aminochrome. Whether aminochrome is actively transported into the nucleus to exert its effects directly or indirectly remains to be determined. The results shown in this study suggest the accumulation of altered proteins that are being ubiquitinated but not degraded by the nuclear proteasome, possibly due to impaired proteasomal activity or an overload that surpasses its degradative capacity. The presence of these aggregates likely reflects cumulative oxidative stress that may compromise nuclear proteins and other macromolecules. Different patterns of nuclear ubiquitin accumulation were observed, ranging from large aggregates to smaller and highly intense ones. Whether ubiquitin labeling corresponds to regions associated with nucleolar function, euchromatin-rich domains, or epigenetic regulatory regions remains to be determined. Consistent with this, previous studies have shown that aminochrome disrupts the cellular redox balance [63] and could generate oxidative damage to lipids and proteins. Aminochrome has also been observed to form adducts and generate oxidative damage to DNA [25,64]. Furthermore, early alterations in gene expression have been reported following aminochrome exposure [23]. Whether oxidative damage occurs early, generating changes in DNA and favoring epigenetic modifications, is unknown. The early upregulation of ubiquitin and LC3B suggests the activation of compensatory mechanisms. Further studies encompassing protein expression levels would be necessary for a better understanding of these mechanisms. Nonetheless, it has been reported that oxidative stress triggers can disrupt normal epigenetic regulation, leading to the dysregulation of transcription factors such as nuclear factor erythroid 2-related factor 2 (NRF2) [65,66]. Interestingly, NRF2 not only regulates genes involved in antioxidant defense, such as glutathione S-transferase Mu 1 (GSTM1) and NAD(P)H quinone dehydrogenase 1 (NQO1), but also orchestrates the expression of autophagy- and proteasome-related genes [67,68,69,70,71,72].

## 5. Conclusions

Our findings suggest that aminochrome-induced disruption of the microtubule network contributes to impaired autophagosome–lysosome fusion and consequent protein aggregate accumulation. Early microtubule destabilization may act as an initiating event, triggering a cascade of microtubule-dependent dysfunctions that contribute, directly or indirectly, to cell death, where protein aggregation may represent both cytotoxic and adaptive (non-toxic) responses.

Notably, the presence of nuclear protein aggregates supports the notion that nuclear proteotoxic stress may play a significant role of aminochrome-induced neurotoxicity. Aminochrome-induced oxidative stress likely represents a key upstream trigger of these events. Whether such nuclear alterations affect proteins involved in nucleolar function, euchromatin-rich domains, or epigenetic regulation remains unknown and warrants further investigation.

In conclusion, therapeutic strategies aimed at preserving microtubule integrity and enhancing proteostasis pathways, including autophagy and the ubiquitin–proteasome system (UPS), may offer novel opportunities for intervention in Parkinson’s disease, particularly in light of emerging evidence linking nuclear proteotoxicity to disease etiology.

## Data Availability

Data are contained in the Supplementary Materials.

## Supplementary Materials

The following supporting information can be downloaded at: https://www.mdpi.com/article/10.3390/antiox15060739/s1, Table S1: Numerical data corresponding to Figure 1B; Table S2: Numerical data corresponding to Figure 1D; Table S3: Numerical data corresponding to Figure 1E; Table S4: Numerical data corresponding to Figure 2B; Table S5: Numerical data corresponding to Figure 3B; Table S6: Numerical data corresponding to Figure 4A; Table S7: Numerical data corresponding to Figure 4B; Table S8: Numerical data corresponding to Figure 4C.

## Abbreviations

The following abbreviations are used in this manuscript:

PD	Parkinson’s disease
LC3β	Microtubule-associated protein 1 light chain 3 beta
LAMP1	Lysosome-associated membrane glycoprotein 1
PI3K	Phosphatidylinositol 3-kinase
AKT	RAC-gamma serine/threonine-protein kinase
mTOR	Serine/threonine-protein kinase mTOR
GAPDH	glyceraldehyde-3-phosphate dehydrogenase
V-ATPases	Vacuolar-type H+-ATPases
SERCA	ATPase sarcoplasmic/endoplasmic reticulum Ca^2+^ transporting
UPS	ubiquitin–proteasome system
