# Supplementary material for: Aminochrome-Induced Disruption of Autophagosome-Lysosome Fusion: Implications for Protein Aggregation in Parkinson’s Disease
Source: Antioxidants (Basel). 2026 Jun 10;15(6):739. doi: 10.3390/antiox15060739 (PMC13295424; doi:10.3390/antiox15060739)
Supplement: Supplementary file 1 [file antioxidants-15-00739-s001.zip › antioxidants-4155452-supplementary.pdf]

Supplementary material

Supplementary Table S1. Numerical data corresponding to Figure 1B.

	Rep 1	Rep 2	Rep 3	Mean	SEM
C	0.55	0.55	0.60	0.57	0.02
AM	0.42	0.42	0.40	0.41	0.01
V	0.30	0.35	0.35	0.33	0.02
AM+V	0.22	0.27	0.14	0.21	0.04
R	0.70	0.69	0.68	0.69	0.01
AM+R	0.31	0.26	TE	0.29	0.02
V+R	0.20	0.34	0.21	0.25	0.04
T	0.70	0.68	0.74	0.71	0.02
AM+T	0.38	0.30	0.45	0.38	0.04
V+T	0.25	0.23	0.20	0.23	0.01

Statistical significance was assessed using analysis of variance (ANOVA) for multiple comparisons and the Newman–Keuls test (**P < 0.01 and ***P < 0.001 compared with control; ♦P < 0.05 and ♦♦♦P < 0.001 compared among experimental groups differing from control). The experiment was performed in three independent replicates, except in group AM+R due to technical error (TE).

Supplementary Table S2. Numerical data corresponding to Figure 1D.

	Rep 1	Rep 2	Rep 3	Mean	SEM
C	465.40	473.80	518,75	485.98	16.56
AM	975.75	800.67	869.50	881.97	50.92
B	1,272.50	1,292.50	1,002.00	1,189.00	93.68
AM+B	1,135.50	984.75	728.17	949.47	118.90

Statistical significance was assessed using analysis of variance (ANOVA) for multiple comparisons and the Newman–Keuls test (**P < 0.01 compared with control). The experiment was performed in three independent replicates.

Supplementary Table S3. Numerical data corresponding to Figure 1E.

	Rep 1	Rep 2	Rep 3	Mean	SEM
C	1.6	1.6	0.7	1.3	0.3
AM	48.0	44.3	53.2	48.5	2.6
V	11.4	12.9	16.0	13.4	1.4
AM+V	53.5	59.8	62.9	58.7	2.8
R	0.3	1.5	2.2	1.3	0.6

AM+R	34.9	37.6	40.8	37.8	1.7
V+R	6.9	8.0	13.4	9.4	2
T	0.4	1.9	2.2	1.5	0.6
AM+T	25.7	20.5	22.8	23.0	1.5
V+T	14.9	20.7	16.4	17.3	1.7

Statistical significance was assessed using analysis of variance (ANOVA) for multiple comparisons and the Newman–Keuls test (***P < 0.001 compared with control; ♦♦♦P < 0.001 compared among experimental groups differing from control). The experiment was performed in three independent replicates.

Supplementary Table S4. Numerical data corresponding to Figure 2B.

	Rep 1	Rep 2	Rep 3	Rep 4	Mean	SEM
C	12.5	25.0	9.1	8.3	13.7	3.9
AM	80.0	90.0	75.0	72.7	79.4	3.8

Statistical significance was assessed using unpaired t-test for two-group comparisons (***P < 0.001 compared with control). The experiments were performed in four independent replicates.

Supplementary Table S5. Numerical data corresponding to Figure 3B.

	Rep 1	Rep 2	Rep 3	Mean	SEM
C	0.520	0.552	0.551	0.54	0.01
AM	0.639	0.571	0.602	0.60	0.02

Statistical significance was assessed using unpaired t-test for two-group comparisons (*P < 0.05 compared with control). The experiments were performed in three independent replicates.

Supplementary Table S6. Numerical data corresponding to Figure 4A.

	Rep 1	Rep 2	Rep 3	Rep 4	Rep 5	Rep 6	Mean	SEM
C	1.16	1.18	1.75	0.65	0.97	1.02	1.12	0.15
AM30	0.5	1	0.27				0.59	0.22
AM4	4.74	3.02	9.48				5.75	1.93
AM9	1.92	0.3	0.52				0.91	0.51
AM14	0.51	1.19	1.12				0.94	0.22

Statistical significance was assessed using analysis of variance (ANOVA) for multiple comparisons and the Newman–Keuls test (***P < 0.001 compared with control). The experiment was performed in three to six independent replicates.

Supplementary Table S7. Numerical data corresponding to Figure 4B.

	Rep 1	Rep 2	Rep 3	Rep 4	Rep 5	Rep 6	Mean	SEM
C	1.00	1.22	0.73	1.77	0.48	0.16	0.89	0.23
AM30	0.02	0.00	0.04				0.02	0.01
AM4	12.42	3.11	TE				7.77	3.80
AM9	0.17	0.20	0.00				0.12	0.06
AM14	0.75	0.66	1.08				0.83	0.13

Statistical significance was assessed using analysis of variance (ANOVA) for multiple comparisons and the Newman–Keuls test (***$P < 0.001$ compared with control). The experiment was performed in three to six independent replicates, except in group AM4 due to technical error (TE).

Supplementary Table S8. Numerical data corresponding to Figure 4C.

	Rep 1	Rep 2	Rep 3	Rep 4	Rep 5	Rep 6	Mean	SEM
C	1.09	1.00	1.0	0.21	0.98	1.02	0,88	0,14
AM30	0.01	2.48	0.03				0,84	0,82
AM4	1.04	0.80	TE				0,92	0,1
AM9	0.01	0,01	0.82				0,28	0,27
AM14	0.04	0.05	TE				0,05	0.01

Statistical significance was assessed using analysis of variance (ANOVA) for multiple comparisons and the Newman–Keuls test. The experiment was performed in three to six independent replicates, except in group AM4 and AM14 due to technical error (TE).
